# Diversity and selection of the continuous-flowering gene, *RoKSN*, in rose

**DOI:** 10.1038/s41438-021-00512-3

**Published:** 2021-04-01

**Authors:** Vanessa Soufflet-Freslon, Emilie Araou, Julien Jeauffre, Tatiana Thouroude, Annie Chastellier, Gilles Michel, Yuki Mikanagi, Koji Kawamura, Mark Banfield, Cristiana Oghina-Pavie, Jérémy Clotault, Alix Pernet, Fabrice Foucher

**Affiliations:** 1 Univ Angers, Institut Agro, INRAE, IRHS, SFR QUASAV, F-49000 Angers, France, 49071 Beaucouzé, France; 2 Natural History Museum and Institute, Chiba, Japan; 3 Osaka Institute of Technology, Osaka, Japan; 4 Department of Biological Chemistry, John Innes Centre, Norwich Research Park, Norwich, UK; 5 Univ Angers, CNRS, TEMOS, SFR CONFLUENCES, Angers, F-49000 France

**Keywords:** Plant breeding, Flowering

## Abstract

Blooming seasonality is an important trait in ornamental plants and was selected by humans. Wild roses flower only in spring whereas most cultivated modern roses can flower continuously. This trait is explained by a mutation of a floral repressor gene, *RoKSN*, a *TFL1* homologue. In this work, we studied the origin, the diversity and the selection of the *RoKSN* gene. We analyzed 270 accessions, including wild and old cultivated Asian and European roses as well as modern roses. By sequencing the *RoKSN* gene, we proposed that the allele responsible for continuous-flowering, *RoKSN*^*copia*^, originated from Chinese wild roses (*Indicae* section), with a recent insertion of the *copia* element. Old cultivated Asian roses with the *RoKSN*^*copia*^ allele were introduced in Europe, and the *RoKSN*^*copia*^ allele was progressively selected during the 19th and 20th centuries, leading to continuous-flowering modern roses. Furthermore, we detected a new allele, *RoKSN*^*A181*^, leading to a weak reblooming. This allele encodes a functional floral repressor and is responsible for a moderate accumulation of *RoKSN* transcripts. A transient selection of this *RoKSN*^*A181*^ allele was observed during the 19th century. Our work highlights the selection of different alleles at the *RoKSN* locus for recurrent blooming in rose.

## Introduction

Flowering is a complex trait under the control of endogenous and environmental factors that allows plants to adapt to different environments and to flower at an appropriate time. Molecular and genetic networks have been deciphered in the model plant *Arabidopsis thaliana*^1^ and progressively in numerous cultivated plants such as rice and cereals^2^, poplar^3^, and pea^4^. Flowering time is an important adaptive trait for plants since the date of flowering is essential for reproductive success, and consequently, for the production of flowers or fruits and the survival of the species. During the processes of natural selection and domestication, flowering has been subjected to major modifications to adapt to different environments and human uses. Evolutionary studies have demonstrated the central role played by flowering genes in these processes. In *A. thaliana*, in the response to vernalization (cold requirement to accelerate flowering), two loci, *FRIGIDA (FRI)* and *FLOWERING LOCUS C (FLC)*, were subjected to natural selection. In the last few thousand years, two nonfunctional alleles at the *FRI* locus were selected, leading to early-flowering ecotypes (no vernalization requirement), probably linked to the weediness of *A. thaliana*^5^. At the *FLC* locus, in the presence of an active *FRI* allele, local adaptation was demonstrated by a latitudinal distribution of haplotypes^6^ that mainly presented *cis* polymorphism, leading to functional differences in the vernalization response during the domestication of rice (*Oryza sativa* L.). Early-flowering and reduced-photoperiod-sensitive genotypes were selected to permit the extension of cultivation to northern latitudes^7^. Nonfunctional alleles at the *Hd1* (*HEADING DATE 1*) locus, a *CONSTANS* homologue, may have been selected to diversify flowering times^8^. Other loci were also proposed for selection in rice^2^. In soybean (*Glycine max*), mutations in a homologue of the floral repressor *TFL1* (*TERMINAL FLOWER 1*) that determines growth habit were selected during domestication^9^. Similarly, in sunflower (*Helianthus annuus*), a dominant mutation in a floral activator *FT* (*FLOWERING LOCUS T*) homologue was selected during domestication, leading to late flowering cultivars via interaction with another *FT* paralog^10^.

In rose (*Rosa* sp.), the processes of natural selection and domestication are poorly understood. Today, rose is the most economically important plant worldwide, with the production of cut, garden and potted roses in the ornamental sector, and the production of essential oils in the perfume sector^11,12^. The tremendous success of rose is due to its symbolism and esthetics. Indeed, throughout the centuries, roses have been chosen for their form, their flower shape and especially their scent. In Europe, rose was already used in ancient Rome. Pliny the Elder described more than six cultivated species thought to correspond to cultivated forms of *R. alba, R. gallica, R. damascena*, and *R. moschata*^13^. Rose cultivation continued in the Middle Ages, not only for its ornamental uses but also as a religious symbol and for its medicinal benefits^14,15^. In Europe, before the 19th century, cultivated roses were mainly hardy once-flowering (OF) shrubs that flowered in spring or early summer. Only a few roses had the ability to occasionally and weakly rebloom in autumn, including *R. moschata* and *R. dasmascena* ‘Quatre saisons’, also known as the Autumn Damask rose^16^. In Asia, roses were cultivated before the Han Dynasty, more than 2000 years ago, with progressive selection of roses with new traits such as continuous-flowering (CF)^17^. The CF trait was only found in cultivated roses. In China, CF roses were first described during the 12th century^17^. At the end of the 18th century, the introduction of Asian roses in Europe caused a revolution in rose breeding with the introduction of new traits such as CF, new scents and new colors^18,19^. A clear genetic differentiation was detected between old European and Asian roses^20^. The 19th century was an intensive period of rose breeding, with crosses between old European and Asian roses^21^ and with a temporal shift from European to Asian genetic background in the hybrids obtained in Europe at that time^20^. During this century, CF behavior was highly selected, leading to the production of CF roses, which were key for the tremendous success of modern roses.

**Fig. 1:**
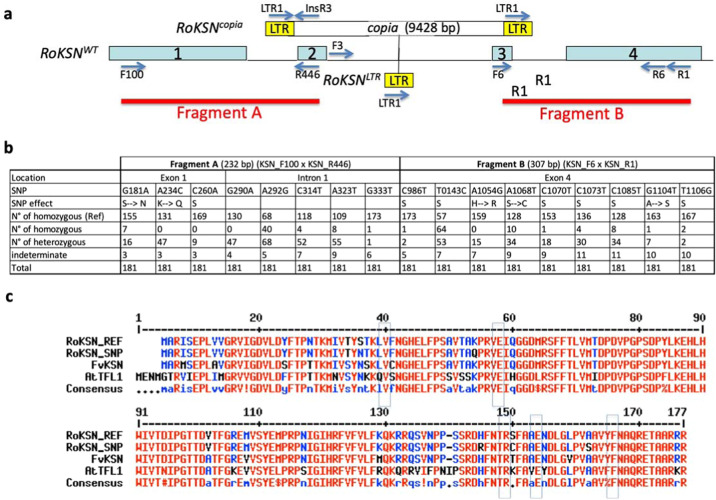
Detection of different alleles at the *RoKSN* locus. **a** Detection of previous alleles^24^: the *RoKSN*^*WT*^ allele encodes a floral repressor homologue to *TFL1*. The gene presents four exons (blue boxes) and three introns (black lines). The *RoKSN*^*copia*^ allele presents the insertion of a *copia* retrotransposon in the 2nd intron. The *RoKSN*^*LTR*^ allele, due to the recombination of the *copia* element, presents only the insertion of the LTR (Long Terminal Repeat) in the 2nd intron. The location and orientation of the primers used to genotype these alleles are represented by arrows. Two PCR fragments (A and B, red lines) were sequenced in order to detect SNPs. Primers are described in Supplementary Table 2. The diagram is not to scale. **b** Frequency of the SNPs detected by Sanger sequencing of the A and B PCR fragments. The SNPs are encoded as follows: G181A means that the SNP at position 181 has a G in the reference sequence (*R*. x *wichurana*) and the A is the variant allele. When the SNP is non-synonymous, the change of the amino acid is indicated. “N° of homozygous (Ref)” is the number of individuals homozygous with the base present in the reference sequence (HQ174211); “N° of homozygous” is the number of individuals homozygous with the variant base; “N° of heterozygous” is the number of individuals heterozygous for the SNP; and “indeterminate” is the number of individuals with missing data. **c** Amino acid alignment of TFL1 homologue protein from rose (RoKSN, HQ174211), strawberry (FvKSN, HQ378595) and *Arabidopsis thaliana* (AtTFL1, AT5G03840.1). RoKSN_REF is the protein encoded by the reference rose sequence and RoKSN_SNP is a hypothetical protein that combines all the non-synonymous changes detected. The non-synonymous changes are surrounded by a blue box

CF behavior is controlled by a monogenic recessive locus^22^. It was recently proposed that two recessive loci might be involved in CF control^23^. Mutation of the floral repressor *RoKSN*, a *TFL1* homologue, was shown to be responsible for the CF phenotype^24^. The recessive mutation leading to CF is due to the insertion of a *copia*-like retrotransposon at the *RoKSN* locus; this allele was designated as *RoKSN*^*copia*^ whereas the dominant allele without this element was designated as *RoKSN*^*WT*^ (Fig. 1a). Due to the insertion of the retrotransposon, the floral inhibitor is no longer accumulated and the rosebush flowers continuously. Interestingly, the *copia*-like retrotransposon can recombine to give a new allele that presents only the LTR (Long Terminal Repeat) element (allele referred to as *RoKSN*^*LTR*^*;* Fig. 1a). This hypomorphic allele is responsible for occasional reblooming in climbing rose mutants^24^. Recently, a null allele (loss of the *RoKSN* gene, referred to as *RoKSN*^*null*^) was detected, and the loss of the gene is due to a large 5-Mbp rearrangement on chromosome 3^25^. Furthermore, in a CF *R. rugosa*, a seasonal expression of *RoKSN* was associated with CF behavior^26^, but the genetic determinism of this CF behavior has not yet been elucidated.

Our objectives were to study the origin, the diversity and the selection of the *RoKSN* gene that controls CF in rose. In Europe, and especially in France, the golden age of rose breeding was the 19th century, so we focused on accessions from this period, which have been preserved by vegetative multiplication in rose gardens.

## Results

### Progressive selection of the *RoKSN*^*copia*^ allele during the breeding process in Europe

In order to study the diversity and selection of the CF locus (*RoKSN*) in rose during the 19th and 20th centuries, we genotyped a large collection of 270 rose accessions for the presence of the *copia* retrotransposon. This large collection of garden roses represented wild Asian and European roses, old cultivated Asian and European roses (assumed to be the ancestors of modern roses), and modern roses (from the late 19th century to the present) (Supplementary Table 1).

Five genotypic profiles were observed at the *RoKSN* locus (Supplementary Table 1): homozygous for the wild allele *RoKSN*^*WT*^ (77 individuals) or for the *RoKSN*^*copia*^ allele (53 individuals), and heterozygous *RoKSN*^*copia*^*/RoKSN*^*LTR*^ (five individuals), *RoKSN*^*copia*^*/RoKSN*^*WT*^ (128 individuals), and *RoKSN*^*copia*^*/RoKSN*^*LTR*^*/RoKSN*^*WT*^ (one individual). All the individuals with the *RoKSN*^*LTR*^ allele were climbing mutants, as previously described^24^. Even if the rose collection presented different ploidy levels, the copy number of each allele (*RoKSN*^*copia*^, *RoKSN*^*LTR*^, and *RoKSN*^*WT*^) was not determined given that they were scored as co-dominant (presence/absence) because of technical limitations.

**Fig. 2:**
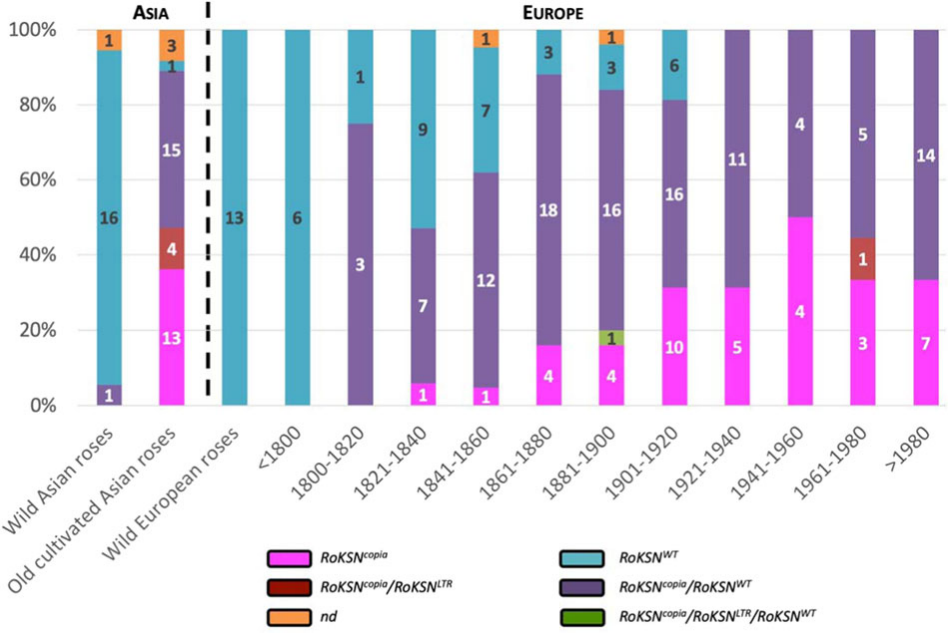
Evolution of the frequency of the different *RoKSN* alleles (*RoKSN*^*WT*^, *RoKSN*^*copia*^, and *RoKSN*^*LTR*^) in Asian and European roses over time. Wild roses were classified according to their geographical origin (Asia or Europe). Cultivated roses were classified according to their breeding date: one class for the old cultivated Asian roses and different temporal classes for cultivated European roses. The breeding dates were manually determined by analysis of historical sources

We studied the frequency of these different genotypic profiles in roses cultivated in Europe during the 19th and 20th centuries (Fig. 2). The Asian and European wild roses presented the allele *RoKSN*^*WT*^ in the homozygous state for the most part (16 out of 18 and 13 out of 13 for Asian and European wild roses, respectively). For the Asian wild roses, one piece of data was missing, and one individual (*R. gigantea* from the rose garden of Haÿ-les-Roses) was *RoKSN*^*WT*^/*RoKSN*^*copia*^. Three *R. gigantea* accessions were present in the studied rose collection, and the other two only had the *RoKSN*^*WT*^ allele. The presence of the *RoKSN*^*copia*^ allele in this assumed wild accession may be explained by a misidentification in the rose garden.

The allele *RoKSN*^*copia*^ was present for most of the old cultivated Asian roses (32 out of 33 accessions with genotyping data; Fig. 2) either in the heterozygous state (15 accessions with *RoKSN*^*copia*^ and *RoKSN*^*WT*^, and four accessions with *RoKSN*^*copia*^ and *RoKSN*^*LTR*^) or in the homozygous state (13 accessions with only *RoKSN*^*copia*^). For European cultivated roses, we observed a gradual decrease of the homozygous genotype profile for the wild allele *RoKSN*^*WT*^ until its absence after the 1920s. On the contrary, the *RoKSN*^*copia*^ allele was increasingly present over time, initially in the heterozygous state (*RoKSN*^*copia*^/*RoKSN*^*WT*^) and then in the homozygous state (*RoKSN*^*copia*^). Indeed, before the 1800s, all European cultivated roses were homozygous for the wild allele *RoKSN*^*WT*^. Between 1800 and 1860, the wild allele was almost equally present in the homozygous and heterozygous states. Then, up until the 1920s, the homozygous profile for the wild allele *RoKSN*^*WT*^ decreased to 15%, whereas the heterozygous profile *RoKSN*^*copia*^/*RoKSN*^*WT*^ increased or remained stable (55–75%). In contrast, the *copia* allele in the homozygous state (*RoKSN*^*copia*^) increased to 30% during this period. After the 1920s, European cultivated roses always presented the *RoKSN*^*copia*^ allele, with an increase in the homozygous state until the 1980s (increasing from 30 to 50%). In the last decades, the cultivated varieties were generally heterozygous (65%) for the *RoKSN*^*copia*^ allele. Surprisingly, some of these heterozygous genotypes (*RoKSN*^*WT*^*/RoKSN*^*copia*^) had the ability to rebloom (Supplementary Table 1), suggesting that the recessive model for the *RoKSN*^*copia*^ allele may be wrong.

These results show a progressive selection of accessions presenting the *RoKSN*^*copia*^ allele at the heterozygous state (1800–1860), then at the homozygous state (1860–1980) and, later, a return to the heterozygous state (1980 to the present).

### The *RoKSN*^*copia*^ allele is a recent allele from Asian wild accessions

The CF behavior of modern roses is considered to originate from old cultivated Chinese roses^17^. The CF behavior is due to an insertion of a *copia*-like retrotransposon into the floral repressor gene *RoKSN*. As we previously showed, the *RoKSN*^*copia*^ allele was present in the large majority of old cultivated Asian roses but was absent in European roses cultivated before 1800 (Fig. 2).

We first investigated the time of insertion of the *copia* element into the *RoKSN* locus. The *copia* element is a LTR (Long Terminal Repeat) retrotransposon, and it was proposed that the insertion time of this retrotransposon type can be estimated by the divergence between the two LTRs at both ends^27^ because their sequences were identical at the insertion event^28^. Assuming that two LTRs accumulate point mutations independently, the nucleotide divergence between the two LTRs reflects the time since the insertion event. We sequenced the 5′ and 3′ LTR elements in *R*. x *wichurana*. The two LTRs were totally similar (Supplementary Fig. 1). The same results were obtained using LTR sequences from the recently released genome sequence (Supplementary Fig. 1). This means that the *copia* element was inserted into the *RoKSN* locus not long ago.

Then, to identify the origin of the *RoKSN*^*copia*^ allele responsible for the CF behavior, we explored the genetic diversity in wild and cultivated rose genetic resources (181 accessions, Fig. 1). We partially sequenced *RoKSN*: two PCR products were sequenced (232 and 307 bp long; Fig. 1a, b). Due to the difficulties to detect insertions/deletions (especially at the heterozygous state), the InDels were not considered. We were able to detect 35 SNPs. Eighteen SNPs were removed because they were rare (less than two occurrences at the heterozygous or homozygous state; Fig. 1 and Supplementary Table 1). Among the 17 remaining SNPs, five were present in the second intron (none were detected in the sequenced part of the third intron). For the 12 SNPs detected in exons, seven were synonymous whereas five were non-synonymous. The most present SNPs (compared to the reference rose sequence^25^) were A292G and T1043C, which were detected in 108 and 117 individuals, respectively, either at the homozygous (40 and 64 accessions for A292G and T1043C, respectively) or the heterozygous (68 and 53 accessions for A292G and T1043C, respectively) state.

**Fig. 3:**
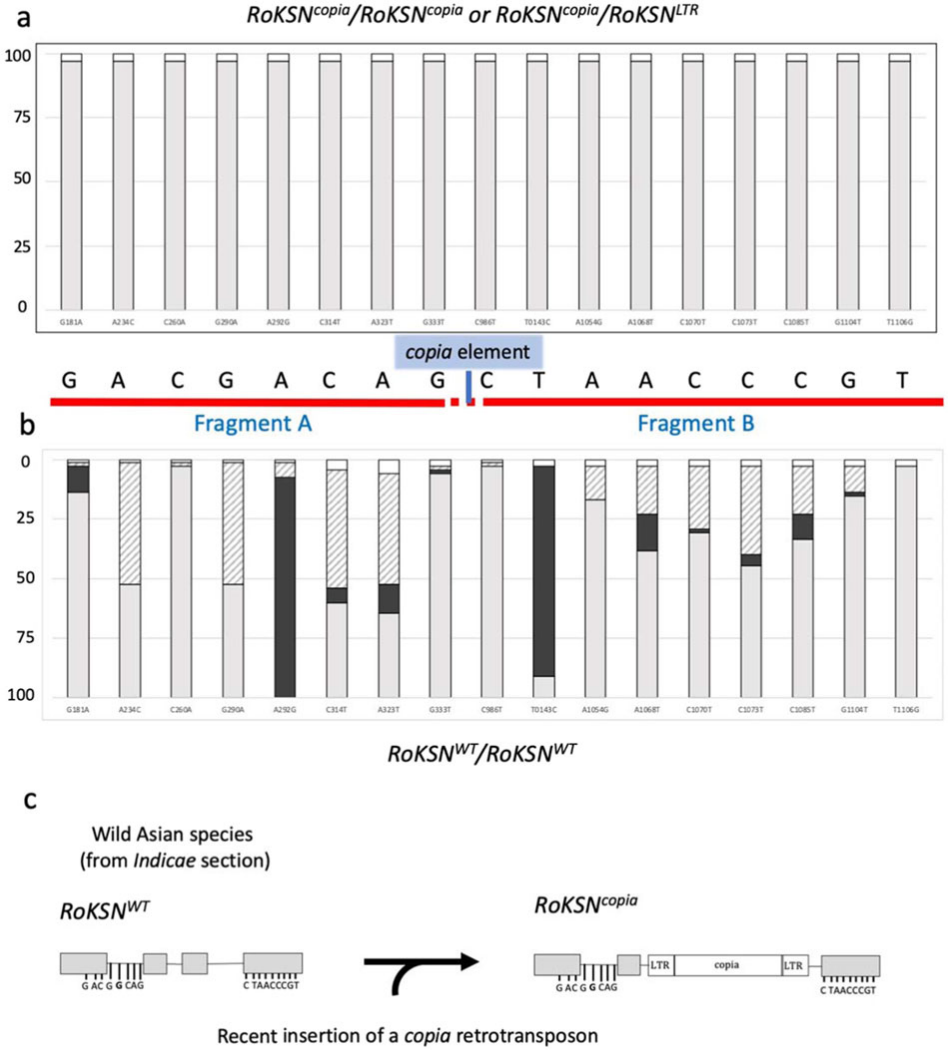
Origin of the *RoKSN*^*copia*^ allele based on the SNP analysis (Fig. 1b). Alleles present in rose accessions (**a**) homozygous *RoKSN*^*copia*^*/RoKSN*^*copia*^ or heterozygous *RoKSN*^*copia*^/*RoKSN*^*LTR*^, or (**b**) homozygous *RoKSN*^*WT*^*/RoKSN*^*WT*^. For each SNP, the proportions of the three possible combinations are presented: homozygous for the allele present in the reference sequence (in gray), homozygous for the alternative allele (in black), heterozygous (in hatched); missing data are in white. (**c**) The *copia* retrotransposon would have recently been inserted into the *RoKSN* gene in rose from the *Indicae* section (*R. chinensis* var. *spontanea* and *R. odorata* var *gigantea*)

Because of technical limitations, allele phases could not be determined. First, we considered only rose accessions that were homozygous *RoKSN*^*copia*^ or heterozygous *RoKSN*^*copia*^/*RoKSN*^*LTR*^. Among the 181 accessions sequenced for the *RoKSN* gene, 34 presented one of these two profiles. All of the 34 accessions (except for one with missing data) presented a single haplotype from the 17 identified SNP sites at the *RoKSN* locus: GACGGCAGCTAACCCGT (Fig. 3a). We assumed that the recent insertion of the *copia* element took place in this haplotype. We then looked at the individuals that presented both this haplotype and the *RoKSN*^*WT*^ allele at the homozygous state in our collection. We found five accessions corresponding to three *R. chinensis* var *spontanea* and two *R. odorata* var *gigantea* accessions (Supplementary Table 1). It should be observed that the two most discriminant SNPs were A292G and T1043C (Fig. 3b). For A292G, the A and G versions were mainly associated with *RoKSN*^*WT*^ and *RoKSN*^*copia*^ alleles, respectively. Similarly, for T1043C, the C and T versions were mainly associated with *RoKSN*^*WT*^ and *RoKSN*^*copia*^ alleles, respectively. Based on these results we proposed that the *copia* retrotransposon might have been inserted into a Chinese wild background (Fig. 3c).

### A new allele at the *RoKSN* locus is associated with recurrent blooming

In order to detect new alleles involved in flowering, we focused on the five non-synonymous SNPs previously detected at the *RoKSN* locus (Fig. 1c). TFL1/FT proteins are known to form a complex, the florigen activation complex FAC^29^. In rice, this heterohexamer complex is composed of two FT proteins, two 14-3-3 proteins and two FD transcription factors. In rose, we assumed that the changes observed in the *RoKSN* gene can modify the activity/structure of the coded protein and can be associated with recurrent blooming.

Among the five non-synonymous SNPs, two (A1068T and G1104T) were thought to have no effect on the protein structure. The former (A1068T) changed a serine into a cysteine, which is a surface residue, independent of any interaction in the florigen activation complex. Furthermore, this amino acid was not conserved in TFL1 proteins (Fig. 1c). The latter (G1104T), resulting in a change of an alanine into a serine, was buried in the structure, and was predicted to have no effect on this structure. This change was present at the homozygous state in ‘Madame Hardy’, which flowered only once in late spring (Supplementary Fig. 2a), suggesting that this modification was not important for the mode of flowering. These two SNPs were not further investigated. Concerning G181A, the change, a serine into an asparagine, is in a residue very close to the 14-3-3 protein in the florigen activation complex and could impact protein:protein interactions (although it is not a direct contact). The A234C SNP (lysine into glutamine) would not be expected to affect the structure with respect to conformation/stability. However, the residue is oriented towards the 14-3-3 proteins in the florigen activation complex and could conceivably alter the interaction. For A1054G, the change (serine into cysteine) is a surface residue independent of any interaction in the florigen activation complex and would not be expected to affect the structure with respect to conformation/stability. Nevertheless, this amino acid was previously described as a major contributor to the difference between TFL1 and FT in *Arabidopsis thaliana*^30^.

We further analyzed these three SNPs by looking for an association between their presence and the blooming pattern. Concerning A234C, we were able to detect the C allele only at the heterozygous state (A/C) in 47 accessions (Fig. 1b and Supplementary Table 1). The same situation was observed for A1054G SNP, where the G allele was only found at the heterozygous state (A/G) in 15 accessions. Among these heterozygous genotypes, we found one wild *R. gallica* for which no recurrent blooming was ever described^18^ and some old cultivated European roses that were OF (such as ‘Tuscany Superb’, ‘Camaieux’, ‘Leda’, and ‘Pergolèse’; Supplementary Table 1 and Supplementary Fig. 2a, c). On the basis of these data, it is difficult to deduce a possible involvement of these SNPs in CF control as they were never found at the homozygous state.

Using sequencing, the A allele of the G181A SNP was found at the heterozygous (16) and homozygous (7) states (Fig. 1b). Among the seven homozygous accessions, four were scored for their blooming pattern and presented the ability to rebloom late in the season and could be considered as recurrent blooming (Supplementary Fig. 2b). After the first flowering period (in spring), new waves of blooming were observed in summer (mainly for two different individuals of *R. fedtschenkoana*) or in summer and autumn for ‘Adam Messerich’ and *R. moschata* (Supplementary Fig. 2b). None of these roses presented the *RoKSN*^*copia*^ or *RoKSN*^*LTR*^ allele (Supplementary Table 3) that could explain their ability to rebloom, as previously proposed^24^. In addition, this A allele at the homozygous state was found in *R. rugosa*, as previously shown^26^. *R. rugosa* is also a recurrent bloomer. The A allele of the G181A SNP is therefore associated with recurrent blooming. We designated this new allele as *RoKSN*^*A181*^. The *RoKSN*^*A181*^ allele of *R. rugosa* and *R. moschata* may share a single genetic origin. The sequence alignment of a whole *RoKSN* gene showed that the *RoKSN*^*A181*^ alleles of *R. rugosa* and *R. moschata* were 99.8% identical (two SNPs per 1080 bp) and grouped into the same cluster (Supplementary Fig. 4).

To genotype this SNP in a larger number of accessions, we developed a dCAPS marker (see Materials and methods) and looked for the presence of the A allele in more recent accessions. We detected 17 accessions with the A allele at the homozygous state and 18 with both alleles, G and A (Supplementary Table 3). Interestingly, the A allele was mainly present during the 19th century (mostly at the heterozygous state; Supplementary Fig. 3). At the heterozygous state, the A allele was mainly present in combination with the *RoKSN*^*copia*^ allele (Supplementary Table 3). After 1900, the A allele was still present in smaller quantities in *Rugosa* hybrid accessions (Supplementary Table 1 and Supplementary Fig. 3).

We assumed that this new *RoKSN* allele might bring about recurrent blooming in rose. In order to test this hypothesis, we investigated this allele in more detail by functional analysis in *Arabidopsis thaliana*.

### The *RoKSN*^*A181*^ transcript is differentially accumulated after the first blooming

We first tested if the protein encoded by the *RoKSN*^*A181*^ allele was functional since the structural analysis suggested that the modification at the protein level could affect the protein-protein interaction in the FAC complex. We performed the complementation of *Arabidopsis thaliana tfl1* mutants with the *RoKSN*^*G181*^ or *RoKSN*^*A181*^ allele. The only difference between these two *RoKSN* alleles was the G or A at position 181 (see Materials and methods). Both alleles were able to complement the *tfl1-11* mutant. The *RoKSN*^*G181*^ allele was already shown to be able to fully complement the *tfl1-11* mutant with indeterminate growth and late flowering (Table 1)^31^. Similar results were obtained with the *RoKSN*^*A181*^ allele. We obtained nine independent lines with indeterminate growth (no terminal flower was observed) and late flowering (from nine to 29 rosette leaves before bolting; Table 1). There were no major differences between the two alleles: *RoKSN*^*G181*^ *and RoKSN*^*A181*^ encoded a functional protein (complementation of the *tfl1* mutants), and the variation did not significantly modify the activity of the protein. In conclusion, like for the *RoKSN*^*G181*^ allele, the protein encoded by the *RoKSN*^*A181*^ allele is active and is a floral repressor in *Arabidopsis*.

**Table 1:** Phenotype of *Arabidopsis thaliana tfl1-11* mutants that ectopically expressed the *RoKSN*^*G181*^ and *RoKSN*^*A181*^ alleles

Genotype	Transformation	N° of rosette leaves	Terminal flower	Seeds	N° of plants
WT (Columbia)	NT	10.8 ± 1.9	–	+	32
*tfl1-11*	NT	5.6 ± 1.0	+	+	32
*tfl1-11_T2*	*RoKSN*^*G181*^	11.0 ± 2.4	–	+	27
	*RoKSN*^*A181*^*_7*	9	–	+	1
	*RoKSN*^*A181*^*_9*	9	−	+	1
	*RoKSN*^*A181*^*_10*	11	−	+	1
	*RoKSN*^*A181*^*_2*	12	–	+	1
*tfl1-11_T1*	*RoKSN*^*A181*^*_6*	13	−	+	1
	*RoKSN*^*A181*^*_3*	14	−	+	1
	*RoKSN*^*A181*^*_4*	15	−	+	1
	*RoKSN*^*A181*^*_8*	24	−	+	1
	*RoKSN*^*A181*^*_1*	29	−	+	1

The phenotype was observed at the T1 generation. The number of rosette leaves was counted. For the terminal flower, “+” means that the inflorescence was rapidly terminated by a flower, “−” means that the inflorescence had an indeterminate growth. The phenotypes of four plants were validated at the T3 generation (Supplementary Table 4)

*NT* not transformed

**Fig. 4:**
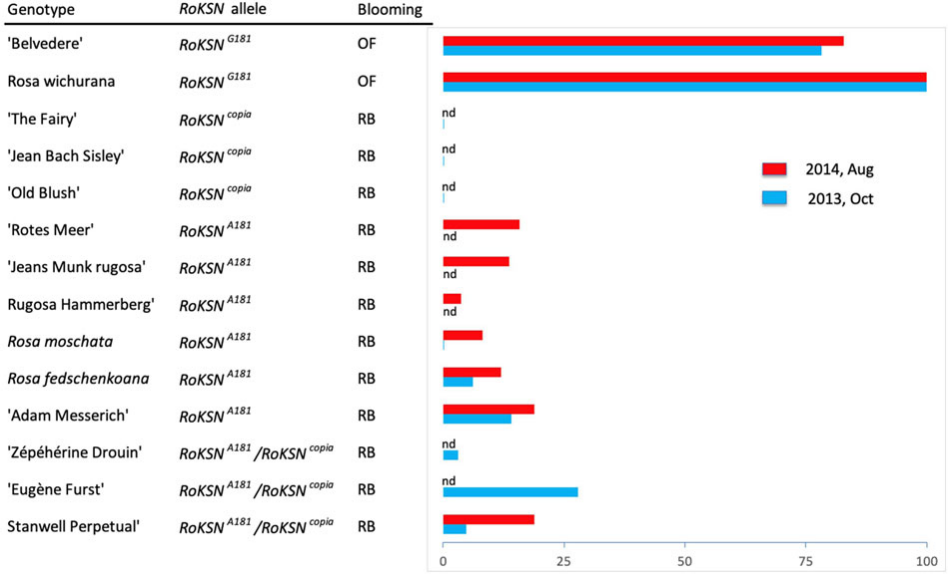
Relative accumulation of the *RoKSN* transcripts in axillary buds after the first blooming in different garden roses: with the *RoKSN*^*copia*^ allele (‘The Fairy’, ‘Jean Bach Sisley’, and ‘Old Blush’) or the *RoKSN*^*G181*^ allele (‘Belvedere’ and *R.* x *wichurana*) or the *RoKSN*^*A181*^ allele (‘Rotes Meer’, ‘Jeans Munk Rugosa’, ‘Moje Hammarberg’, *R. moschata*, *R. fedschenkoana*, and ‘Adam Messerich’). Transcript accumulation was evaluated by q-PCR and expressed relatively to *R*. x *wichurana* (value 100) (nd: not determined). The mode of blooming was determined based on the flowering pattern on Supplementary Fig. 2, except for ‘The Fairy’, ‘Jean Munk Rugosa’, ‘Moje Hammarberg’, ‘Zéphérine Drouin’, ‘Eugène Furst’, and ‘Stanwell Perpetual’, where it was based on the information of helpmefind (https://www.helpmefind.com/rose/plants.php). OF for once-flowering and RB for recurrent blooming

We then tested if the expression of the *RoKSN*^*A181*^ allele was not modified during the reblooming process. We compared the *RoKSN* transcript accumulation in roses homozygous for the *RoKSN*^*A181*^, *RoKSN*^*G181*^ or *RoKSN*^*copia*^ allele. As previously demonstrated^24^, roses presenting only the *RoKSN*^*copia*^ allele did not accumulate *RoKSN* transcripts (‘Old Blush’, ‘The Fairy’, and ‘Jean Bach Sisley’; Fig. 4), and they flowered continuously (Supplementary Fig. 2). The roses with the *RoKSN*^*G181*^ allele accumulated *RoKSN* transcripts in large amounts (*R*. x *wichurana* and ‘Belvédère’; Fig. 3) and were OF (Supplementary Fig. 2). The roses with the *RoKSN*^*A181*^ allele (‘Adam Messerich’, *R. fedtschenkoana, R. moschata*, ‘Moje Hammarberg’, ‘Jeans Munk’, and ‘Rotes Meer’) still accumulated *RoKSN* transcripts in comparison with CF roses, but this accumulation was 10–20-fold less than for OF roses (Fig. 4). As previously shown, these roses had the ability to rebloom later in the season, but not continuously (Supplementary Fig. 2). Furthermore, we also studied the transcript accumulation in three roses (‘Eugène Furst’, ‘Zéphérine Drouin’, and ‘Stanwell Perpetual’) that had the *RoKSN*^*A181*^ and *RoKSN*^*copia*^ alleles. These recurrent blooming roses presented a low *RoKSN* transcript accumulation, largely weaker than in OF roses, demonstrating again that the *RoKSN*^*A181*^ allele brings a low *RoKSN* transcript accumulation.

## Discussion

### Several alleles for recurrent blooming

Rose presents different modes of flowering, ranging from roses that flower only in spring (OF) to roses that can flower all the time as long as the growing conditions are favorable (CF)^32^. We previously demonstrated that the loss of function of the floral repressor *KSN*, a *TFL1* homologue, is responsible for the CF phenotype^24^. Two mutations were detected at the *RoKSN* locus. One is due to the insertion of a large *copia* transposable element in the second intron (*RoKSN*^*copia*^ allele)^24^; the other allele (*RoKSN*^*null*^) is due to a 5 Mb rearrangement, leading to the loss of several genes, including *RoKSN*^25^. Interestingly, many roses have intermediate phenotypes with a weaker ability to rebloom, considering the blooming quantity and period (such as *R. fedschenkoana* and ‘Adam Messerich’; Supplementary Fig. 2). For instance, climbing mutants with a weak ability to rebloom were shown to present a recombinant *RoKSN*^*copia*^ allele. This recombinant allele presents only the LTR element in the second intron (*RoKSN*^*LTR*^)^24^.

In this work, we identified a new allele at the *RoKSN* locus that might be responsible for recurrent blooming. Using sequencing, we identified 17 SNPs at this locus, with five SNPs leading to protein modifications (Fig. 1). Among these five non-synonymous SNPs, one (G181A) was responsible for the change of a serine into an asparagine; the A version of this SNP at the homozygous state was associated with recurrent blooming. Almost all the plants (15 out of 17) homozygous for the A allele were able to rebloom. Genetic data, such as a cross between these plants and a heterozygous plant, should be necessary to conclude if the *RoKSN* locus is responsible for this recurrent blooming. Given that the SNP can affect the protein structure (non-synonymous SNP), we had assumed that the protein activity could be modified. However, on the basis of functional validation in *Arabidopsis thaliana* (*tfl1* mutant complementation), we were unable to detect any changes between the proteins encoded by the G or A allele (Table 1), demonstrating that the protein was still active as a floral repressor. We cannot exclude that minor modifications in the activity could not be detected due to the fact that the gene was expressed ectopically under the control of a strong promoter (35S). Another hypothesis is that the transcriptional regulation of the allele is modified and the *RoKSN*^*A181*^ modification is not the causal modification but a modification linked with other variants in the regulatory elements. We clearly showed that the plants with the A allele accumulated less transcripts than the plants with the G allele (Fig. 4). We hypothesize that the recurrent blooming observed for the plants with the A allele is due to a weaker accumulation of the floral inhibitor. Other associated SNPs (located perhaps in the regulatory regions as promoters or introns) might be responsible for differences in transcript accumulation. Further investigations are necessary to clearly identify the causal modifications. Sequences in the second intron are good candidates since the presence of a LTR element in this intron (Climbing mutants)^24^ also leads to plants with weak reblooming capacity. In pea, a similar transcriptional regulation was observed for *LATE FLOWERING*, another *TFL1* homologue. Different alleles were detected with different levels of expression: low expression was associated with early-flowering whereas high expression was associated with late flowering^33^.

The genetic determinism of recurrent blooming seems to be more complicated that previously proposed. CF was first described as being controlled by a recessive mutation of a floral repressor in F1 progenies^22,24^. With this recessive hypothesis, we expected that the heterozygous genotypes, *RoKSN*^*copia*^/*RoKSN*^*WT*^, would be OF. However, we observed lots of genotypes (96 out of 117) that were able to rebloom with different intensities (Supplementary Table 1). These results suggest that the recessive hypothesis may be wrong. Other elements should be taken into account as: (1) other loci can interfere with the *RoKSN* locus as proposed^23^, (2) other alleles can be present at the *RoKSN* locus which were not considered, as the *RoKSN*^*null*^ allele^25^, (3) we should consider the dose of the allele in a polyploid context which is known to modify the expression of the phenotype as shown in *Brassica* for flowering date^34^, and (4) we cannot exclude that the *RoKSN*^*copia*^ allele can interfere with the *RoKSN*^*WT*^ allele by modifying its expression as described for the paramutation where one allele (paramutagenic) can silence the other allele (paramutated) with a high variability in the penetrance and heritability^35^. Further analyses should be necessary to verify these hypotheses.

### Scenario of *RoKSN* allele selection

Blooming is an important breeding criterion for garden roses. Until the 19th century, European roses were mostly non-recurrent, blooming only in the spring^19^. Only some species like *R. moschata* were able to rebloom in the summer or autumn. Nevertheless, blooming for these European roses was lower than for Asian roses with CF. This CF behavior is due to an insertion of a *copia*-like retrotransposon in the *RoKSN* floral repressor gene^24^. Our results obtained for the *RoKSN* locus suggest the following scenario.

We showed that the *copia* element was always associated with a particular haplotype (Fig. 3). In the absence of the *copia* element, this haplotype was only found in wild Chinese species from the *Indicae* section: *R. chinensis* var. *spontanea* and *R. odorata* var. *gigantea*^36^. This suggests that the *copia*-like retrotransposon was inserted into a wild Chinese species or an old cultivated rose from a wild Chinese species, which are known to produce flowers only in spring (OF behavior). When a transposable element (such as *copia*) is inserted, it creates a pair of LTRs with identical sequences at the two breakpoints^28^. At the *RoKSN* locus, both LTRs of the *copia* element are identical (Supplementary Fig. 1), demonstrating that the *RoKSN* mutation (i.e., the retrotransposon insertion in *RoKSN*) occurred recently. This scenario is strengthened by the presence of only one haplotype associated with the *RoKSN*^*copia*^ allele (Fig. 3). We can assume that this mutation was selected by the Chinese and more widely used in Asian rose breeding (as proved by the presence of the *copia* element in the old cultivated Asian roses; Fig. 2) to obtain CF roses. It should be noted that we cannot exclude the selection of other alleles of Asian background. For example, a null allele was identified in *R. chinensis* ‘Old Blush’ at the *RoKSN* locus, but this allele cannot be followed because of the loss of the *RoKSN* gene^25^.

Later, before the 19th century, these Asian CF mutants were introduced into Europe^18^ and then crossed with OF European roses and, to a lesser degree, with European occasional-reblooming roses, to give rise to modern roses, which flowered many times a year. In the 19th and 20th centuries, it is then assumed that by selecting roses increasingly reblooming, breeders were selecting the *copia* allele (*RoKSN*^*copia*^), which resulted in an increase of the presence of this allele in European cultivated roses, first at the heterozygous state and later at the homozygous state (Fig. 2). Interestingly the same trade was observed with neutral microsatellite markers in another collection of garden roses bred in Europe during the 19th century. Indeed, a progressive shift for the European garden roses from a European genetic background to an Asian one during this century was demonstrated^20^. In our study, we showed that, at the *RoKSN* locus, the allele initially present in European roses, *RoKSN*^*WT*^, was similarly progressively replaced by the Asian-origin allele *RoKSN*^*copia*^, which is responsible for CF. This allele selection was associated with the selection of rose accessions that flowered continuously, which helped the rose to play a prominent role on the European and world markets^37^.

Moreover, our results showed a transient selection of the *RoKSN*^*A181*^ allele during the 19th century (Supplementary Fig. 3). In that century, some rose accessions presented both the *RoKSN*^*A181*^ and *RoKSN*^*copia*^ alleles, which suggests that these roses were obtained from crosses between CF roses (with the *RoKSN*^*copia*^ allele) and occasional-reblooming roses (with the *RoKSN*^*A181*^ allele), where breeders would have combined a hypomorphic allele (*RoKSN*^*A181*^) and a recessive allele (*RoKSN*^*copia*^). It can be suggested that these hybrids (*RoKSN*^*copia*^*/RoKSN*^*A181*^) were more reblooming than hybrids with the *copia* allele at the heterozygous state (*RoKSN*^*copia*^*/RoKSN*^*WT*^). This raises the question of the dose of the different alleles in the polyploid context, which is the case of the rose accessions bred during the 19th century^38^. As we were unable to quantify the different alleles at the *RoKSN* locus, it was not possible to test this hypothesis in our study. The origin of the *RoKSN*^*A181*^ allele is not clear since the allele is found in Asian (such as *R. rugosa*) and European (such as *R. moschata*) materials.

Although the geographical distributions of the two roses are separated^39^, we hypothesize that *R. moschata* has a wild ancestor related to *R. rugosa* (Supplementary Fig. 4). *Rosa fedchenkoana* has the *RoKSN*^*A181*^ allele with the weak reblooming characteristics (Supplementary Fig. 2) and is placed in the section *Cinnamomea* together with *R. rugosa*^36^, and is therefore a putative wild ancestor of *R. moschata*. We speculate that the *RoKSN*^*A181*^ allele originated in wild roses of the section *Cinnamomea*, inherited by *R. moschata* through hybridization, and was artificially selected in the old European accessions.

Our work sheds new light on the selection of genes that control flowering in rose. We clearly demonstrated the selection of an Asian-origin allele during the 19th and 20th centuries in Europe. This selection was associated with the breeding of CF roses and their worldwide success.

## Materials and methods

### Materials

A large collection of garden roses (270 accessions) was sampled. This collection consisted of wild Asian (18 accessions) and European (13 accessions) roses, old cultivated Asian (36 accessions) and European (108 accessions) roses, and modern roses (from the late 19th century to the present; 95 accessions). The studied roses are detailed in Supplementary Table 1. For the cultivated roses, the date when each rose accession was obtained was determined by a historian on the basis of various historical sources. Due to unavailability or discrepancy between the historical sources, the date was declared “unknown” for 45 accessions (Supplementary Table 1). For expression analysis, 11 accessions were selected in the “Loubert” Rose Garden (Rosiers-sur-Loire, France) with contrasting modes of flowering: OF roses (‘Belvédère’ and *Rosa* x *wichurana*); CF roses (‘The Fairy’, ‘Jean Bach Sisley’, and *R. chinensis* ‘Old Blush’); and occasional-reblooming roses (‘Rotes Meer’, ‘Jeans Munk Rugosa’, ‘Moje Hammarberg’, *R. moschata*, *R. fedschenkoana*, and ‘Adam Messerich’).

### Methods

#### Genotyping of the *RoKSN* gene

DNA was extracted using the NucleoSpin Plant II kit (Macherey-Nagel, Düren, Germany) according to the manufacturer’s instructions. The 270 accessions of the rose collection were genotyped for the three known different alleles of *RoKSN: RoKSN*^*WT*^*, RoKSN*^*copia*^ and *RoKSN*^*LTR*^, which were identified using two pairs of primers: KSN_F100 x KSN_R6 and KSN_LTR1 x KSN_R6 (Fig. 1a; Supplementary Table 2). However, these two PCRs did not make it possible to discriminate *RoKSN*^*copia*^/*RoKSN*^*LTR*^, and *RoKSN*^*LTR*^/*RoKSN*^*LTR*^ genotypes. For these genotypes, two additional pairs of primers were used: KSN_F3 × KSN_InsR3 and KSN_F3 × KSN_R1 (Fig. 1a). PCR reactions were performed in 15 µl with 10 ng genomic DNA, 1 X Green GoTaq^®^ Flexi buffer, 0.125 mM dNTPs, 2 mM MgCl_2_, 0.2 µM of each primer, and 0.1 U GoTaq^®^ Flexi DNA polymerase (Promega Corporation, Madison, WI, USA). Amplifications were carried out using MyCycler thermalcycler (Bio-Rad, Inc., Hercules, CA, USA). The thermal cycling protocol had an initial denaturation at 94 °C for 2 min, followed by 40 cycles of denaturating at 94 °C for 45 s, annealing at 60 °C for 45 s and extension at 72 °C for 90 s, and ended with a 7-min final extension at 72 °C. The amplified fragments were separated on a 1.7% agarose gel and then stained with ethidium bromide and revealed by ultraviolet light.

#### Sequencing of the *RoKSN* gene

A partial sequencing of the *RoKSN* gene was performed for 181 accessions (listed in Supplementary Table 1), which mainly corresponded to wild and old cultivated roses. Two PCR fragments were amplified on each side of the *copia* element: fragment A (336 bp, using the KSN_F100 and KSN_R446 primer pair) and fragment B (383 bp, using the KSN_R1 and KSN_F6 primer pair). The PCR conditions used were the same as for the *RoKSN* gene genotyping. PCR products were sequenced using SANGER technology with the same primer pairs used for the PCR amplification (Supplementary Table 2). The sequences were manually checked and were aligned with Geneious software v8.1.8 (Biomatters Ltd., Auckland, New Zealand). Due to difficulties to detect insertions/deletions (especially at the heterozygous state), InDels were not considered. SNPs with less than two occurrences at the heterozygous or homozygous state were removed. Each SNP was labeled by its position on the sequence of *Rosa* x *wichurana* (HQ174211^24^) and the nucleotide polymorphism at this position. For example, SNP A292G means that a SNP was identified at position 292 and that the accessions had either adenine or guanine at this genome position.

#### Development of a dCAPS marker to genotype the polymorphism at the G181A SNP

Degenerated CAPS (dCAPS) was developed to genotype the G181A SNP detected by sequencing. The primers (mos_Pst1_F1 and mos_dcaps_R1; Supplementary Table 2) were designed using dCAPS Finder 2.0^40^. PCR reactions were performed in a volume of 15 µl with 10 ng genomic DNA, 1 X Green GoTaq^®^ Flexi buffer, 0.125 mM dNTPs, 2 mM MgCl2, 0.2 µM of each primer, and 0.1 U GoTaq^®^ Flexi DNA polymerase (Promega Corporation, Madison, WI, USA), with the following program: 95 °C for 3 min, 40 cycles (95 °C for 30 s, 60 °C for 40 s, and 72 °C for 30 s), and 72 °C for 5 min.

Amplified PCR fragments were then digested using *PstI* restriction enzyme (#ER0615, Thermo Fisher Scientific Inc.) according to the manufacturer’s instructions. The digestion led to the production of three DNA fragments for the allele, with a guanine at position 181, *RoKSN*^*G181*^ (37, 50 and 64 bp), and two DNA fragments for the allele with an adenine at the same position, *RoKSN*^*A181*^ (64 and 87 bp). DNA fragments were separated on a Resophor gel (4.5% w/v), stained with ethidium bromide.

#### Genetic transformation of *Arabidopsis thaliana tfl1* mutants with the *RoKSN*^*G181*^ or *RoKSN*^*A181*^ allele

##### Plasmid construction

The *RoKSN*^*G181*^ allele was obtained from a construction previously obtained in a p35:KSN:pENTR plasmid^31^. We used this construction to substitute the G for an A by proceeding to a site-directed mutagenesis using the QuikChange II Site-Directed Mutagenesis Kit (Agilent, Santa Clara, CA, USA), according to the manufacturer’s instructions, with the primers mRmKSN_F2 and mRmKSN-R3 (Supplementary Table 2). The sequences were validated by SANGER sequencing. The two alleles were then cloned into the pK7WG2D destination vector using a GATEWAY LR Clonase II kit (Invitrogen, Carlsbad, CA, USA), according to the manufacturer’s instructions. The ligation products were transferred into One Shot TOP10 Competent *Escherichia coli* (Thermo Fisher Scientific, Waltham, MA, USA) by thermal shock at 42 °C for 1 min.

##### Genetic transformation of Arabidopsis thaliana

*Arabidopsis thaliana* (L.) *tfl1-11* mutants (N6235)^41^ were provided by the Nottingham *Arabidopsis* Stock Center. The binary vector was introduced by electroporation into *Agrobacterium tumefasciens* EHA105^42^ containing the plasmid *pbbR*. *A. thaliana tfl1-11* mutants were transformed using the floral dip method^43^. Transformed plants were selected on Murashige and Skoog basal medium supplemented with 50 mg l^−1^ kanamycin. Plants were grown under long day conditions (16 h: 8 h, light: dark; 20 °C) and were scored for the number of rosette leaves and the presence of terminal flowers.

#### Expression analysis of *RoKSN* alleles in different rose accessions

Young growing shoots after axillary bud outgrowth were harvested in October 2013 and in August 2014 in the “Loubert” Rose Garden (Rosiers-sur-Loire, France). Total RNAs were extracted from axillary buds using the NucleoSpin RNA Plant kit (Macherey-Nagel, Düren, Germany). The absence of genomic DNA contamination was checked by PCR^31^. cDNAs were obtained by reverse transcription performed on 500 ng of total RNA using Iscript Ready-to-use (Bio-Rad, Inc., Hercules, CA, USA). The real-time amplification (q-PCR) was performed with SsoADV Univer SYBR Green Supermix (Bio-Rad, Inc., Hercules, CA, USA) using cDNA as a template, with the following program: 98 °C for 30 s and 40 cycles (98 °C for 10 s, 60 °C for 10 s). Fluorescence detection was performed using a CFX ConnectTM Real-Time System (Bio-Rad, Inc., Hercules, CA, USA). The amount of cDNA in each sample was normalized using the *TCTP* gene^44^, and the relative expression level was calculated according to Pfaffl^45^ from two biological replicates and three technical repetitions per replicate. Primers for the amplification of *RoKSN* and *TCTP* genes are listed in Supplementary Table 2.

#### Dating of the *copia* element insertion

To date the *copia* element insertion in the *RoKSN* locus, we sequenced the two LTR elements from *R*. x *wichurana* using SANGER technology with the following primers: KSN_F3 and KSN_InsR3 for the 3′LTR, and KSN_LTR5′F and KSN_R5 for the 5′LTR. Sequences were analyzed using Geneious software v8.1.8 (Biomatters Ltd., Auckland, New Zealand). For a second genotype, *R. chinensis* ‘Old Blush’, we recovered the LTR sequences from the haploid genome reference^46^ with the *copia* element (ms324231_RchiOBHmChr3_RLC_denovoRcHm_v2.0-B-P313.2180-Map5_reversed) inserted into the *RoKSN* locus (RchiOBHm_Chr3g0473011 and RchiOBHm_Chr3g0473021).

#### Phenotyping of recurrent blooming

Several rose accessions were phenotyped in the “Loubert” Rose Garden (Rosiers-sur-Loire, France) in 2012 and 2013 (‘Adam Messerich’, ‘Belvédère’, ‘Camaieux’, ‘Jean Bach Sisley’, ‘Leda’, ‘Madame Hardy’, ‘Pergolèse’, *R. fendschenkoana*, *R. moschata*, *R*. x *wichurana*, ‘Stanwell Perpetual’, ‘Old Blush’, and ‘Tuscany Superb’). The number of flowers at a particular stage (OFM for open flower, as described^47^) was counted every week for six months (from mid-May to mid-November), and the recurrent blooming was estimated by visual observation (Supplementary Fig. 3).

## Supplementary information

Supplementary Figure 1

Supplementary Figure 2

Supplementary Figure 3

Supplementary Figure 4

Supplementary Table 2

Supplementary Table 3

Supplementary Table 4

Supplementary Table 1

## Data Availability

All the plant material used in this study are described in details in Supplementary Table 1 with the rose gardens where the roses were collected.
