# Supplementary figures and images for: Diversity and selection of the continuous-flowering gene, RoKSN, in rose
Source: Hortic Res. 2021 Apr 1;8:76. doi: 10.1038/s41438-021-00512-3 (PMC8012652; doi:10.1038/s41438-021-00512-3)

## Slide 1
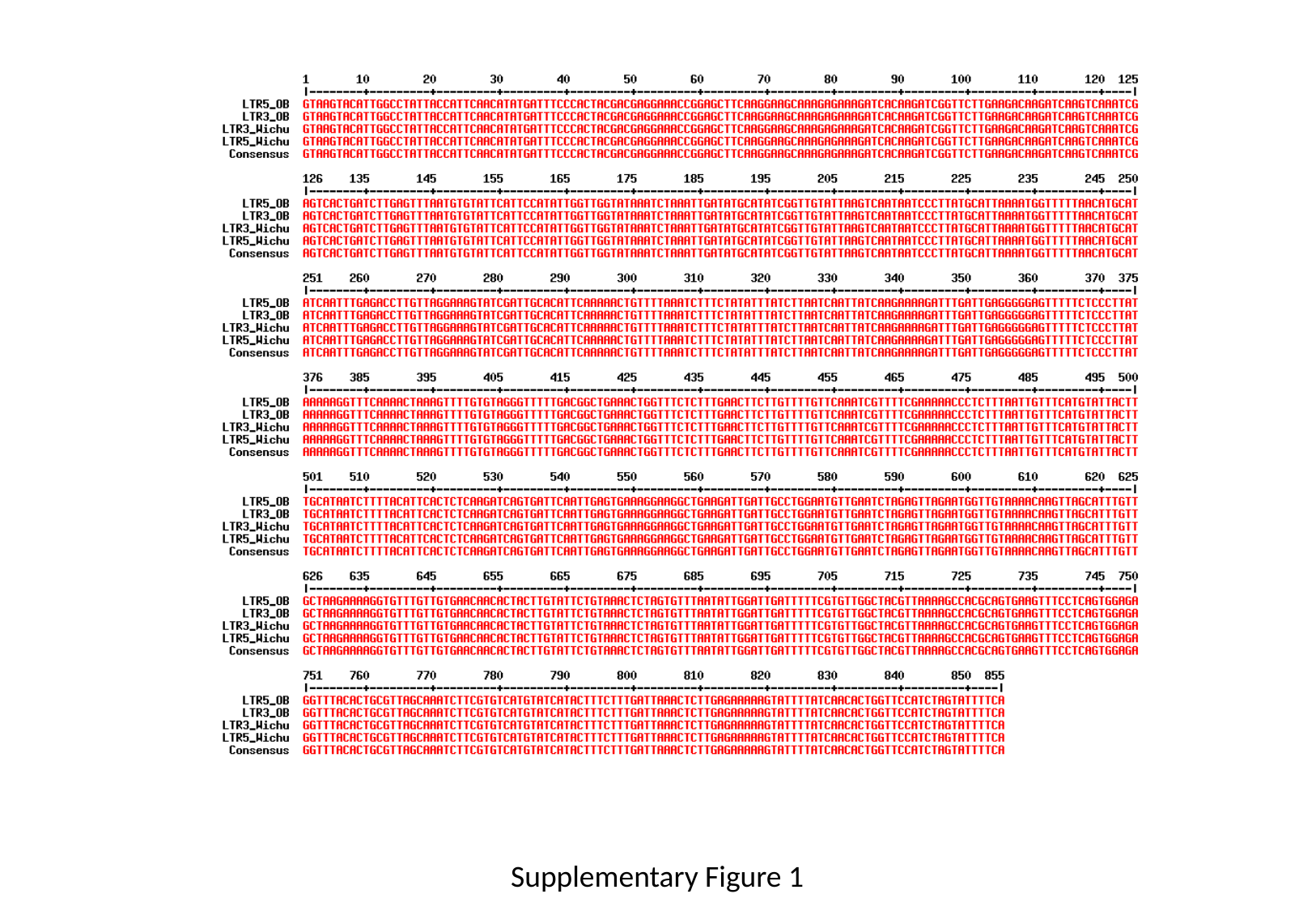

Supplementary Figure 1

Supplement: Supplementary file 1 — Supplementary Figure 1 [file 41438_2021_512_MOESM1_ESM.pptx]

## Slide 1
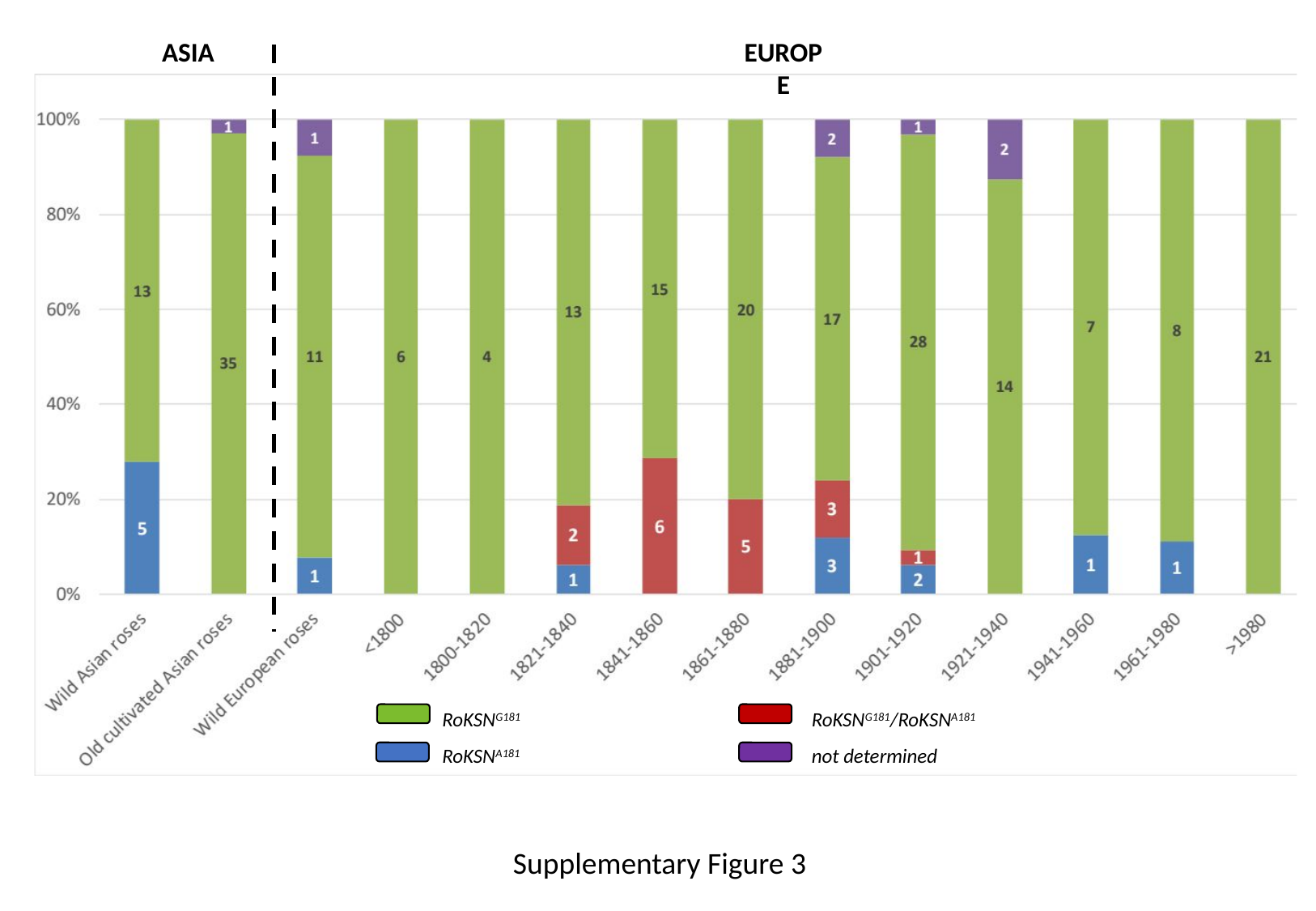

Asia
Europe
RoKSNG181
RoKSNA181
RoKSNG181/RoKSNA181
not determined
Supplementary Figure 3

Supplement: Supplementary file 3 — Supplementary Figure 3 [file 41438_2021_512_MOESM3_ESM.pptx]
