# Supplementary material for: Diversity and selection of the continuous-flowering gene, RoKSN, in rose
Source: Hortic Res. 2021 Apr 1;8:76. doi: 10.1038/s41438-021-00512-3 (PMC8012652; doi:10.1038/s41438-021-00512-3)
Supplement: Supplementary file 2 — Supplementary Figure 2 [file 41438_2021_512_MOESM2_ESM.pptx]

## Slide 1
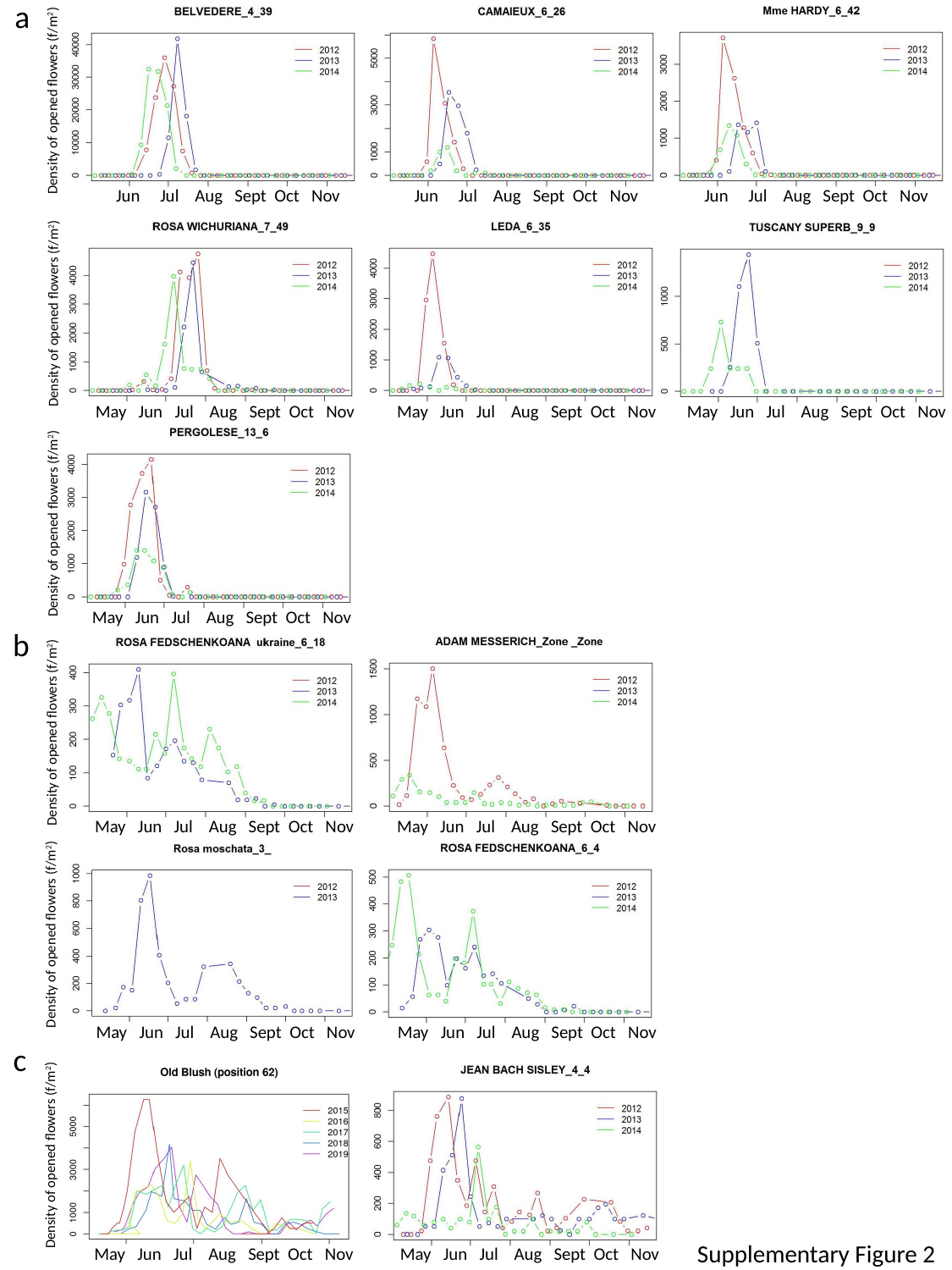

a
Density of opened flowers (f/m2)
Jun Jul Aug Sept Oct Nov
Jun Jul Aug Sept Oct Nov
Jun Jul Aug Sept Oct Nov
Density of opened flowers (f/m2)
May Jun Jul Aug Sept Oct Nov
May Jun Jul Aug Sept Oct Nov
May Jun Jul Aug Sept Oct Nov
Density of opened flowers (f/m2)
May Jun Jul Aug Sept Oct Nov
b
Density of opened flowers (f/m2)
May Jun Jul Aug Sept Oct Nov
May Jun Jul Aug Sept Oct Nov
Density of opened flowers (f/m2)
May Jun Jul Aug Sept Oct Nov
May Jun Jul Aug Sept Oct Nov
c
Density of opened flowers (f/m2)
Supplementary Figure 2
May Jun Jul Aug Sept Oct Nov
May Jun Jul Aug Sept Oct Nov
