# Supplementary material for: Diversity and selection of the continuous-flowering gene, RoKSN, in rose
Source: Hortic Res. 2021 Apr 1;8:76. doi: 10.1038/s41438-021-00512-3 (PMC8012652; doi:10.1038/s41438-021-00512-3)
Supplement: Supplementary file 4 — Supplementary Figure 4 [file 41438_2021_512_MOESM4_ESM.pptx]

## Slide 1
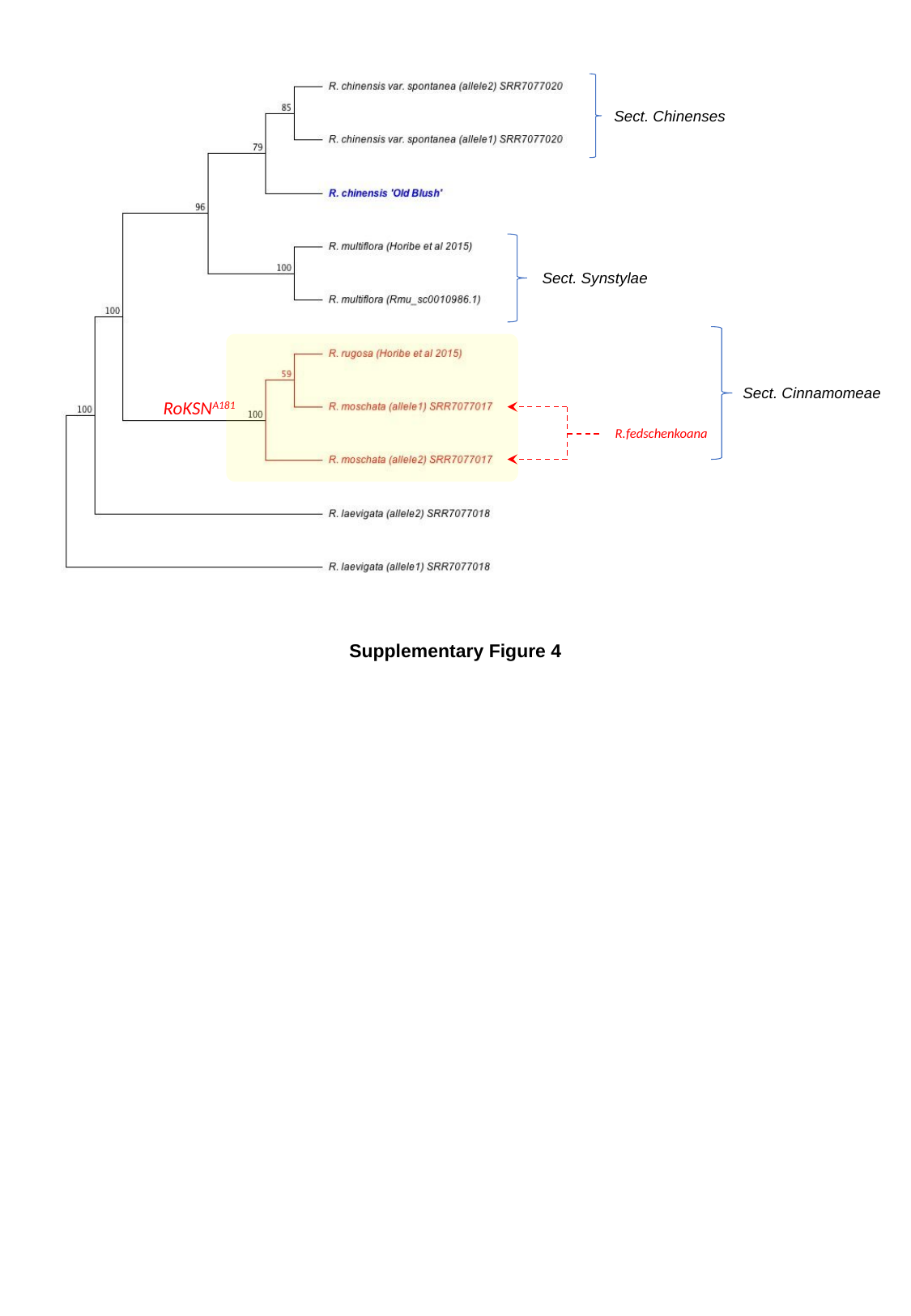

Sect. Chinenses
Sect. Synstylae
Sect. Cinnamomeae
RoKSNA181
R.fedschenkoana
Supplementary Figure 4
